# TAK1 inhibition-induced RIP1-dependent apoptosis in murine macrophages relies on constitutive TNF-α signaling and ROS production

**DOI:** 10.1186/s12929-015-0182-7

**Published:** 2015-09-18

**Authors:** Jang-Shiun Wang, Dean Wu, Duen-Yi Huang, Wan-Wan Lin

**Affiliations:** Department of Pharmacology, College of Medicine, National Taiwan University, No 1, Sec 1, Jen-Ai Road, Taipei, Taiwan; Graduate Institute of Medical Sciences, Taipei Medical University, Taipei, Taiwan; Department of Neurology, Shuang Ho Hospital, Taipei Medical University, New Taipei City, Taiwan

**Keywords:** TAK1, RIP1, Macrophages, TNF-α, ROS, Apoptosis

## Abstract

**Background:**

Transforming growth factor-β (TGF-β)-activated kinase 1 (TAK1) is a key regulator of signal cascades of TNF-α receptor and TLR4, and can induce NF-κB activation for preventing cell apoptosis and eliciting inflammation response.

**Results:**

TAK1 inhibitor (TAKI) can decrease the cell viability of murine bone marrow-derived macrophages (BMDM), RAW264.7 and BV-2 cells, but not dermal microvascular endothelial cells, normal human epidermal keratinocytes, THP-1 monocytes, human retinal pigment epithelial cells, microglia CHME3 cells, and some cancer cell lines (CL1.0, HeLa and HCT116). In BMDM, TAKI-induced caspase activation and cell apoptosis were enhanced by lipopolysaccharide (LPS). Moreover, TAKI treatment increased the cytosolic and mitochondrial reactive oxygen species (ROS) production, and ROS scavengers NAC and BHA can inhibit cell death caused by TAKI. In addition, RIP1 inhibitor (necrostatin-1) can protect cells against TAKI-induced mitochondrial ROS production and cell apoptosis. We also observed the mitochondrial membrane potential loss after TAKI treatment and deterioration of oxygen consumption upon combination with LPS. Notably TNF-α neutralization antibody and inhibitor enbrel can decrease the cell death caused by TAKI.

**Conclusions:**

TAKI-induced cytotoxicity is cell context specific, and apoptosis observed in macrophages is dependent on the constitutive autocrine action of TNF-α for RIP1 activation and ROS production.

## Background

Transforming growth factor-β (TGF-β)-activated kinase 1 (TAK1) is a ubiquitously expressed mitogen-activated protein kinase kinase kinase and plays a key role in regulating inflammation, immunity, cell differentiation and death [1–3]. Accumulating evidence indicates that TAK1 is a key regulator of signal transduction cascades and is activated by various inflammatory mediators and cytokines such as transforming growth factor (TGF)-β, tumor necrosis factor (TNF)-α, interleukin (IL)-1β, CD40 ligand, toll-like receptor (TLR) ligands, T and B cell receptor ligands [3, 4]. TAK1 activity is tightly regulated by its binding proteins, TAB1 and TAB2/TAB3, as well as by post-translational modification including ubiquitination and phosphorylation. In TLR4 signaling, TRAF6 through its E3 ubiquitin ligase activity facilitates the formation of K63 polyubiquitin chains to recruit and activate TAK1 [5]. This activation then transduces signals for activating downstream kinases IKK, p38, and JNK, in turn leading to activate NF-κB and activator protein-1 (AP-1) to produce the proinflammatory and anti-apoptosis proteins [4, 6–8]. Consistently the notion implicating TAK1 as a key intermediary in cell survival is evidenced by observing the embryonic lethality associated with multi-tissue defects in germline deficient of TAK1 gene [4]. Subsequent studies in mice with the conditional ablation of TAK1 in hepatocytes [9], liver parenchymal cells [10], keratinocytes [11], intestinal epithelial cells [12], dendritic cells [13], and monocytes [14], further strengthen the cytoprotection role of TAK1.

TNF-α contributes to many physiological and pathological processes and plays an important role in mediating survival signaling, apoptosis and necroptosis depending on the cell types and cellular context [1]. On TNF-α binding, TNF receptor 1 (TNFR1) undergoes a conformational change to form TNFR complex I containing TRADD, receptor-interacting protein (RIP)1, cIAPs, TRAF2 and TRAF5. RIP1 ubiquitylation by cIAP1 or TRAF2 can recruit the TAK1 to initiate the canonical NF-κB survival pathway [1, 4, 10, 15]. Upon inhibition of NF-κB signaling, for example by de-ubiquitylation of RIP1 and loss of cIAPs, TNFR complex II (TRADD, FADD, caspase 8, RIP1 and RIP3) can be formed and execute two distinct types of cell death, apoptosis and necroptosis that compete for each other and is switched by TAK1 and caspases. Normally, caspase 8 triggers apoptosis by cleaving RIP1 to inhibit the pro-survival actions derived from NF-κB signaling, and the pro-apoptotic protein Bid to generate a truncated form (tBid) for inducing cytochrome c release and apoptosome formation. However, when caspase 8 is blocked by pharmacological or genetic interventions, RIP1 can recruit RIP3 to form the RIP1/RIP3 necrosome. Within this necrosome, RIP1 and RIP3 phosphorylate each other, further stabilizing the complex and engaging the effector mechanisms of necroptosis, which is a recently identified programmed cell death [16, 17]. Necroptosis has been shown to rely on mitochondrial reactive oxygen species (ROS) production and disintegration of mitochondrial, lysosomal and plasma membranes [18–20]. Moreover, necroptosis has been shown to involve in the pathogenesis of various diseases, such as ischemic injury, neurodegeneration, viral infection, liver injury, traumatic brain injury, and severe drug reaction [21].

Notably, recent studies further demonstrate the role of TAK1 in controlling the RIP1-dependent apoptosis triggered by TNFR complex II. Besides inducing NF-κB-dependent anti-apoptotic mechanisms, an NF-κB-independent cell survival pathway downstream of TAK1 and involving the regulation of ROS-cIAPs-RIP1-caspase pathway has been suggested [2, 22, 23]. Moreover, in mouse embryonic fibroblasts (MEF), TAK1 activity was shown to be a switch between apoptosis and necroptosis following TNF-α stimulation [24]. Hyperactivation of TAK1 possibly through inhibition of caspase-1 leads to necroptosis in dermal fibroblasts which results in the delay in wound healing. In contrast, ablation of TAK1 causes RIP1- and caspase-dependent apoptosis in MEF and monocytes [14, 24]. Since TAK1 is a potential therapeutic target of inflammatory disorders and cancer [25], it is essential to more understand the role of TAK1 in cell death regulation in different cell types. Moreover, since the role of TAK1 in controlling RIP1-dependent apoptosis was just recently raised and demonstrated only in limited cell types under certain cellular context (e.g. under TAK deficiency plus treatment with either TNF-α or Smac mimetic), it is warrant to further elucidate the detailed regulatory mechanisms underlying this event. Therefore, in this study using 5Z-7-oxozeaenol (TAKI), which is a natural product of fungal origin and an irreversible TAK1 inhibitor [26], we found a cell type specific action of TAKI in apoptosis, and this action occurring in myeloid cells is dependent on endogenously constitutive TNFR-mediated RIP1 activation and mitochondrial ROS production.

## Methods

### Cell culture

Bone marrow-derived macrophages (BMDM) were isolated from 8–12 week old C57BL/6 mice as we previously described [27]. Lung cancer CL1.0 cell line was provided by Dr. Zhixin Yang (National Taiwan University, Taipei, Taiwan). Murine RAW264.7 macrophages, and other cell lines were obtained from the American Type Culture Collection (Manassas, VA, USA). The normal human epidermal keratinocytes (NHEKs) were prepared as we previously described according to the Mackay Memorial Hospital Institutional Review Board (IRB 12MMHIS193) [28]. The CL1.0, HeLa, BV-2, RAW264.7, J774, A431 and CHME3 cells were cultured in Dulbecco’s modified Eagle’s medium (DMEM). The HCT116 and THP-1 cells were cultured in RPMI 1640 medium. The human retinal pigment epithelial cells (ARPE) were cultured in a 1:1 mixture of DMEM and Ham’s F12 medium. Human dermal microvascular endothelial cells (DMVEC) were cultured in Vascular Cell Basal Medium containing Kit-VEGF (ATCC® PCS100 041™). NHEKs were cultured in Keratinocyte-SFM (Gibco BRL/Invitrogen, Carlsbad, CA) supplemented with recombinant epidermal growth factor (0.1–0.2 ng/ml) and bovine pituitary extract (20–30 mg/ml). All culture media contain 10 % (v/v) heat-inactivated FBS, 100 U/ml penicillin and 100 μg/ml streptomycin. Cells were incubated at 37 °C in a humidified atmosphere of 5 % CO_2_ in air.

### Measurement of cell viability by MTT assay and LDH release

After indicated drug treatment, cells (2 × 10^4^/ml) in 96-well plates were incubated with 1 mg/ml 3-(4,5-dimethylthiazol-2-yl)-2,5-diphenyltetrazolium bromide (MTT) at 37 °C for 60 min. The culture medium was removed, and then the formazan granules generated by live cells were dissolved in DMSO. The net absorbance (OD_550_ - OD_630_) indicating the enzymatic activity of mitochondria and cell viability was measured. In some experiments, cytotoxicity was assessed by measuring the release from damaged cells of the cytosolic enzyme lactic dehydrogenase (LDH). After treating cells with the indicated agents, LDH activity present in the culture medium was determined using a LDH diagnostic kit (Promega, Heidelberg, Germany). The absorbance values were read at OD_490_ on a Synergy HT Multi-Detection Reader (Bio-Tek Instruments, Winooski, VT, USA). Data were expressed as the percentages of total cellular amount of LDH that was determined by lysing cells with lysis buffer (0.9 % Triton X-100).

### Intracellular ATP assay

Intracellular ATP was determined by CellTiter-Glo® Luminescent Kit (G7571, Promega Heidelberg, Germany) according to the manufacturer’s protocol. After indicated drug treatment, medium was replaced with the new one, and the reagent in the kit was added to lyse the cells. The plate was gently shaken for 2 min, placed stationary in the dark for 10 min, and then 150 μl solution was transferred to a 96-well plate for luminescence detection using LB96V MicroLumat plus (American Laboratory Trading, Boston, USA). With background subtraction, the values were normalized to individual control group as 100 %.

### Intracellular ROS detection

After being treated for the indicated time periods, cells were collected and washed with PBS twice, then incubated in PBS containing 5 μM DCFH-DA (for detecting the cytosolic ROS) or MitoSOX red (for detecting superoxide in the mitochondria of live cells) for 30 min at 37 °C. After incubation, cells were immediately subjected to flow-cytometry analysis using the FACScan. The data were analyzed with the CellQuest program.

### Mitochondrial respiration measurement

The oxygen consumption rate (OCR) was measured by the Extracellular flux analyzer XF24 (Seahorse Bioscience, Houston, TX, USA). About 50,000 BMDM cells were plated on laminin-coated XF24 plates and cultured for 24 h in a 5 % CO_2_ incubator at 37 °C. Then, the medium was removed and replaced by 500 μl FX assay medium (143 mM NaCl, 5.4 mM KCl, 0.8 mM MgSO_4_, 1.8 mM CaCl_2_, 0.91 mM Na_2_HPO_4_, 2 mM glutamine, 2 mg/ml BSA, 3 mg/l phenol red, and other constituents, pH 7.4.) The BMDM cells were preincubated for 1 h at 37 °C in normal atmosphere. Stock solutions (×10) of 1 μg/ml LPS, 1 μM TAKI or LPS plus TAKI (injection on port A), 10 μg/ml oligomycin (ATP synthase inhibitor in port B), 2.5 μM carbonyl cyanide-p-trifluoro methoxyphenylhydrazone (FCCP, a mitochondrial uncoupler of oxidative phosphorylation in port C), and 2.5 μM rotenone (an inhibitor of mitochondrial Complex I in port D) were prepared in FX24 assay media and loaded into injection ports respectively, then measurements were obtained at 37 °C.

### Statistical evaluation

Values were expressed as the mean ± S.E.M. of at least three independent experiments, which were performed in duplicate. Analysis of variance was used to assess the statistical significance of the differences, and *P* value < 0.05 was considered statistically significant.

### Ethical approval

The animal experiments were conducted in accordance with institute regulations after receiving approval from the Ethics Committee of the National Taiwan University College of Medicine (No. 20130391).

## Results

### TAKI induced apoptotic cell death in BMDM

**Fig. 1:**
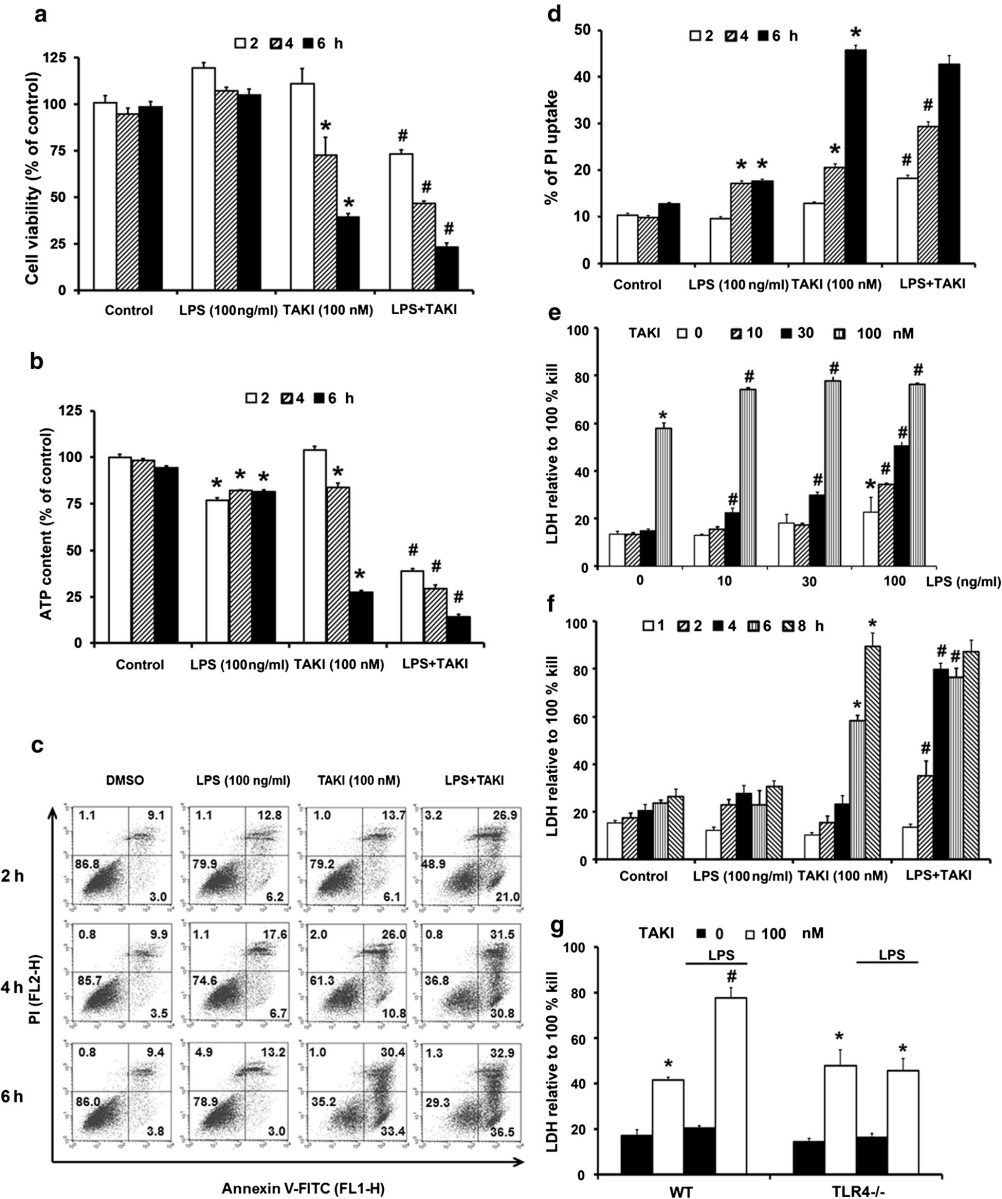
TAKI induced apoptotic cell death in BMDM. Cells were treated with TAKI (100 nM) and/or LPS (100 ng/ml) for 2, 4 and 6 h. Then cell viability assessed by MTT assay (**a**), ATP content assay (**b**), Annexin V/PI staining (**c**) and PI uptake (**d**) was determined. In some experiments for LDH release assay, TAKI and/or LPS at different concentrations were given to BMDM for 6 h (**e**), or treating both to BMDM for different time intervals (**f**). In (**g**), wild type (WT) and TLR4^−/−^ BMDM were treated with TAKI (100 nM), LPS (100 ng/ml) or both for 6 h, and LDH activity was measured. *p <0.05, indicating the significant effect of TAKI or LPS alone on cell death. #*p* <0.05, indicating the significant potentiation of cell death by LPS in TAKI-treated cells

Using MTT assay as the index of cell viability, we found that TAKI (100 nM) treatment for 4 h can induce cell death of BMDM. Since LPS is a potent TAK1 and NF-κB activator in BMDM, we interested to understand whether TAKI-induced cytotoxicity is affected under LPS treatment. Notably we found that LPS at sub-cytotoxic concentration (100 ng/ml) can enhance cell death of TAKI (Fig. 1a). Measuring intracellular ATP level also showed the cytotoxic effect of TAKI and lower ATP content under simultaneous activation of TLR4 (Fig. 1b). To verify the cell death mode, we determined Annexin V and PI staining. Results showed that cell population of Annexin V positive staining, either with or without higher PI staining, was increased along with the time of TAKI treatment (Fig. 1c). In agreement with data of MTT and ATP assays, TAKI-induced increase of Annexin V-positive cell number was elevated in the presence of LPS (Fig. 1c).

Next using PI uptake for necrosis assessment, we found the amount of cells with positive PI staining was increased by TAKI, and was also enhanced in the presence of LPS (Fig. 1d). Further determining the cell necrotic LDH release, we found the concentration- (Fig. 1e) and time- (Fig. 1f) dependent effects of LPS to enhance the TAKI-induced response. Using TLR4^−/−^ BMDM, we confirmed the potentiation effect of LPS on TAKI-induced cell death is resulting from TLR4 activation (Fig. 1g). Given that PI uptake and LDH release were induced along with the increased Annexin V staining, it is suggested that the cell death elicited by TAKI is apoptosis followed by the fast proceeding to necrosis.

### TAKI-induced apoptosis depends on RIP1

**Fig. 2:**
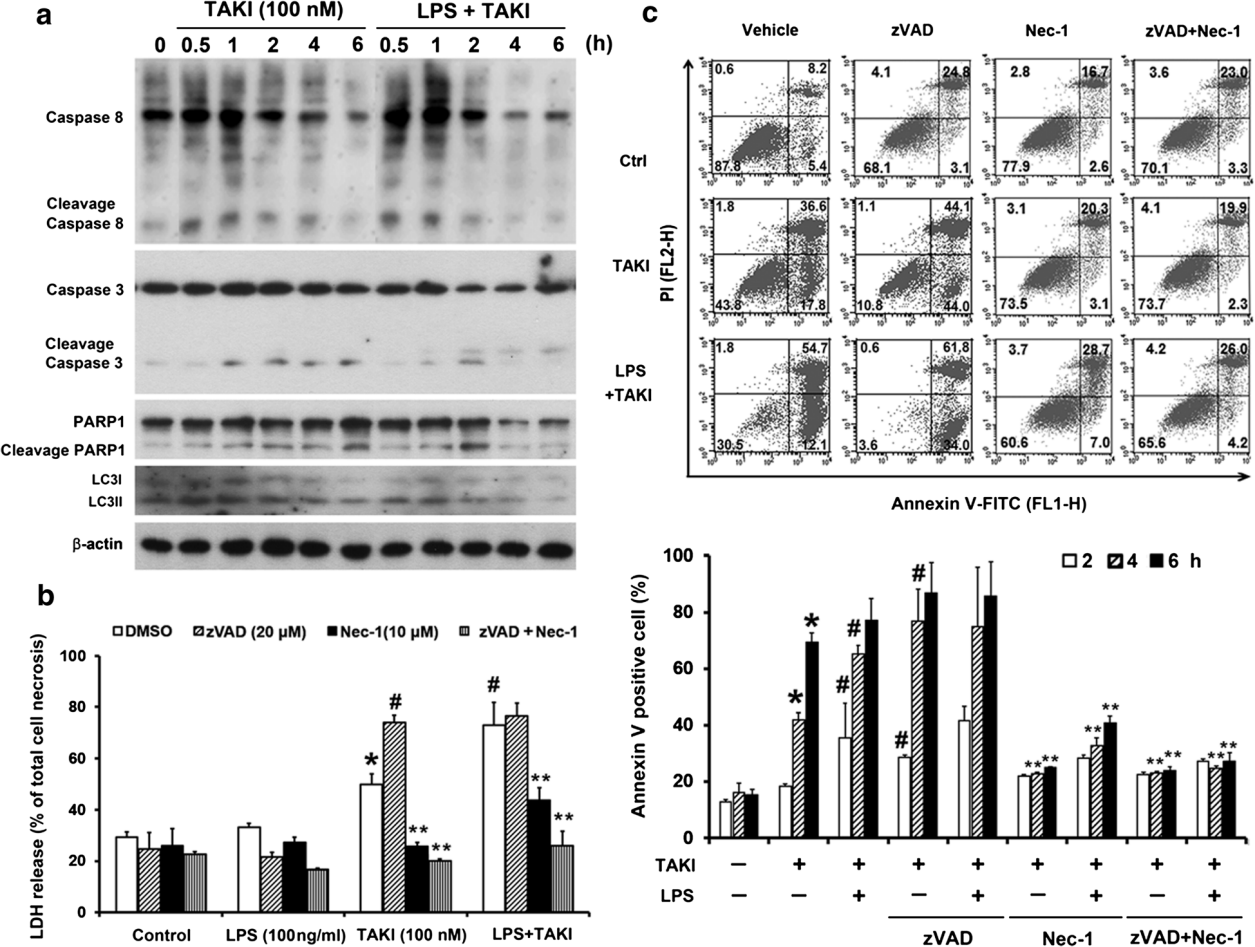
TAKI-induced cytotoxicity is dependent on RIP1 activity. (**a**) BMDM were treated with TAKI (100 nM) and/or LPS (100 ng/ml) for indicated time periods. Cell lysates were collected for immunobloting. (**b**, **c**) BMDM were pre-treated with necrostatin-1 (Nec-1, 10 μM) and/or zVAD (20 μM) for 30 min followed by adding TAKI (100 nM) and/or LPS (100 ng/ml) for 6 h. LDH activity in the collected culture medium (**b**) and amount of Annexin V/PI positive cells (**c**) were determined. *p <0.05, indicating the significant effect of TAKI alone. #*p* <0.05, indicating the significant effects of LPS and zVAD on the response of TAKI. **p <0.05, indicating the significant inhibition of TAKI-induced cell death by Nec-1

Confirming apoptotic feature, TAKI can induce active caspase 8 and caspase 3 formation, and PARP1 cleavage (Fig. 2a). Although LPS co-treatment facilitated the downregulation of pro-caspase 3 and PARP1, it was hard to detect the increased cleavage forms of both proteins. We speculate this is possibly due to the instability of both cleaved proteins. These results suggest that LPS stimulation can enhance TAKI-elicited apoptotic caspase activation. Since TAK1 has been shown to regulate autophagy in breast epithelial cells MCF10A and human cervical carcinoma HeLa cells [29, 30], we wondered whether this event occurs in BMDM. When autophagy is activated, LC3II [LC3-phosphatidylethanolamine (PE)] formation is prerequisite for autophagosome formation and is regarded as an autophagy marker [31]. Results of immunobloting showed no increased effect of TAKI, with or without LPS co-treatment, on LC3II/LC3I ratio (Fig. 2a).

To characterize the cell death pathway in BMDM, we examined the role of RIP1. To this end, we pre-treated cells with 10 μM necrostatin-1 (Nec-1, a selective RIP1 inhibitor) and found that Nec-1 can protect cells from TAKI-induced LDH release (Fig. 2b) and Annexin V staining (Fig. 2c). Such protection was also observed in cells with LPS and TAKI co-treatment. Since non-selective caspase inhibitor zVAD was reported to sensitize macrophages and MEF for RIP1-dependent necroptosis [32–34], we further treated macrophages with zVAD to clarify the cell death effects of TAKI. Results revealed that zVAD itself did not affect cell viability (Fig. 2b, c), but its presence further potentiated the TAKI-induced cytotoxicity. Nevertheless when enhanced cytotoxicity was already induced by TAKI + LPS, zVAD no longer increased the cell toxicity. Moreover, the death induced by TAKI + LPS, no matter in the presence or not of zVAD, was diminished by Nec-1 (Fig. 2b, c). These results suggest the involvement of RIP1 in TAKI-induced cell death regardless of the presence of LPS or zVAD.

### TAKI-induced cytotoxicity depends on ROS

**Fig. 3:**
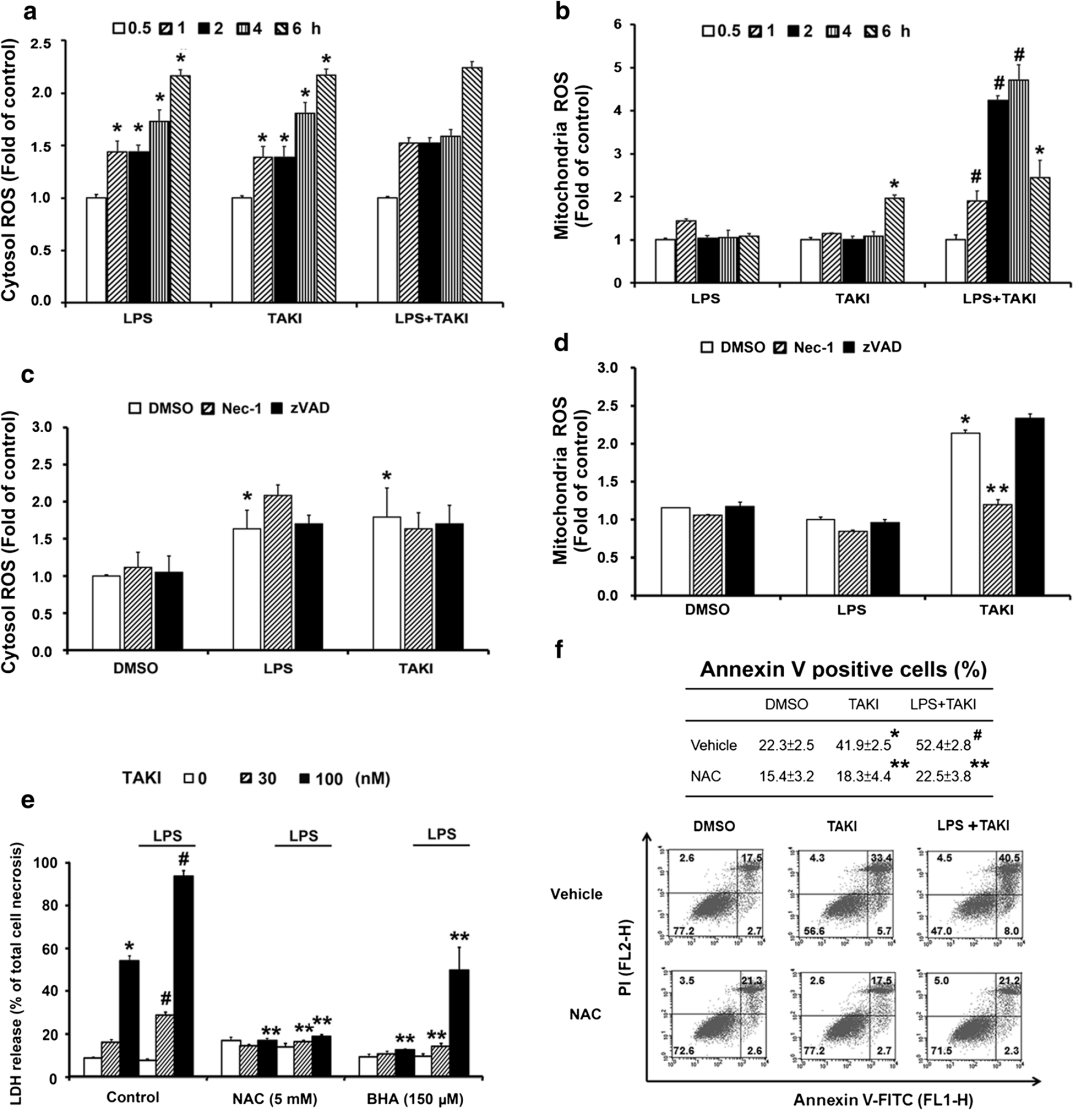
TAKI-induced cytotoxicity is dependent on mitochondrial ROS. (**a**, **b**) BMDM were treated with TAKI (100 nM), LPS (100 ng/ml), or both for indicated time periods. (**c**, **d**) BMDM were pre-treated with Nec-1 (10 μM) or zVAD (20 μM) for 30 min before the addition of TAKI, LPS or both for 6 h. The cytosolic and mitochondrial ROS production were determined by DCFH-DA (**a**, **c**) and MitoSOX (**b**, **d**), respectively, and we used the levels under vehicle treatment as the control. (**e**, **f**) BMDM were pre-treated with NAC (5 mM) or BHA (150 μM) for 30 min followed by TAKI and/or LPS stimulation. After 6 h incubation, LDH release was determined (**e**) and after 4 h drug treatment, Annexin V/PI staining (**f**) was determined. *p <0.05, indicating significant effects of TAKI or LPS alone. #*p* <0.05, indicating the significant potentiation of TAKI-induced responses by LPS. **p <0.05, indicating significant inhibitory effects of Nec-1, NAC and BHA on the responses induced by TAKI, either in the absence or presence of LPS

Since ROS play an important role in apoptosis and necrosis, we used DCFH-DA and MitoSOX staining to determine cytosolic and mitochondrial ROS levels respectively. The data revealed that both TAKI (100 nM) and LPS (100 ng/ml) can increase cytosolic ROS level (Fig. 3a) with similar extent, and their co-treatment was non-additive (Fig. 3a). Compared to the early onset (30 min) of ROS production in cytosol, mitochondrial ROS production was observed at 6 h after TAKI treatment. Although LPS alone did not cause significant mitochondrial ROS production, its co-treatment with TAKI led to an enhancement of mitochondrial ROS production in terms of its onset and extent (Fig. 3b). Due to the apparent cytotoxicity, we did not detect a sustained mitochondrial ROS increase at 6 h in TAKI + LPS group. Accordingly we tested the effects of Nec-1, NAC (a general antioxidant through production of intracellular GSH), BHA (a ROS scavenger) and zVAD on ROS response. Since the onset of TAKI-induced cytosolic ROS production is much earlier than that of mitochondrial ROS, and RIP1-mediated mitochondrial ROS is implicated in necroptotic cell death [35], we wonder if the early increased cytosolic ROS is also mediated by RIP1. As a result, Nec-1 can reduce the mitochondrial but not cytosolic ROS increase induced by TAKI (Fig. 3c, d). On the other hand, zVAD did not cause any changes in this aspect (Fig. 3c, d). In addition, NAC and BHA can markedly reduce LDH release induced by TAKI/LPS (Fig. 3e), and NAC also reduced the amount of Annexin V positive cells in response to TAKI (Fig. 3f). These results suggest the prerequisite role of mitochondrial ROS production triggered by RIP1 rather than cytosolic ROS in TAKI-induced cell death.

### No contribution of IKK and MAPKs to TAKI-induced cell death

**Fig. 4:**
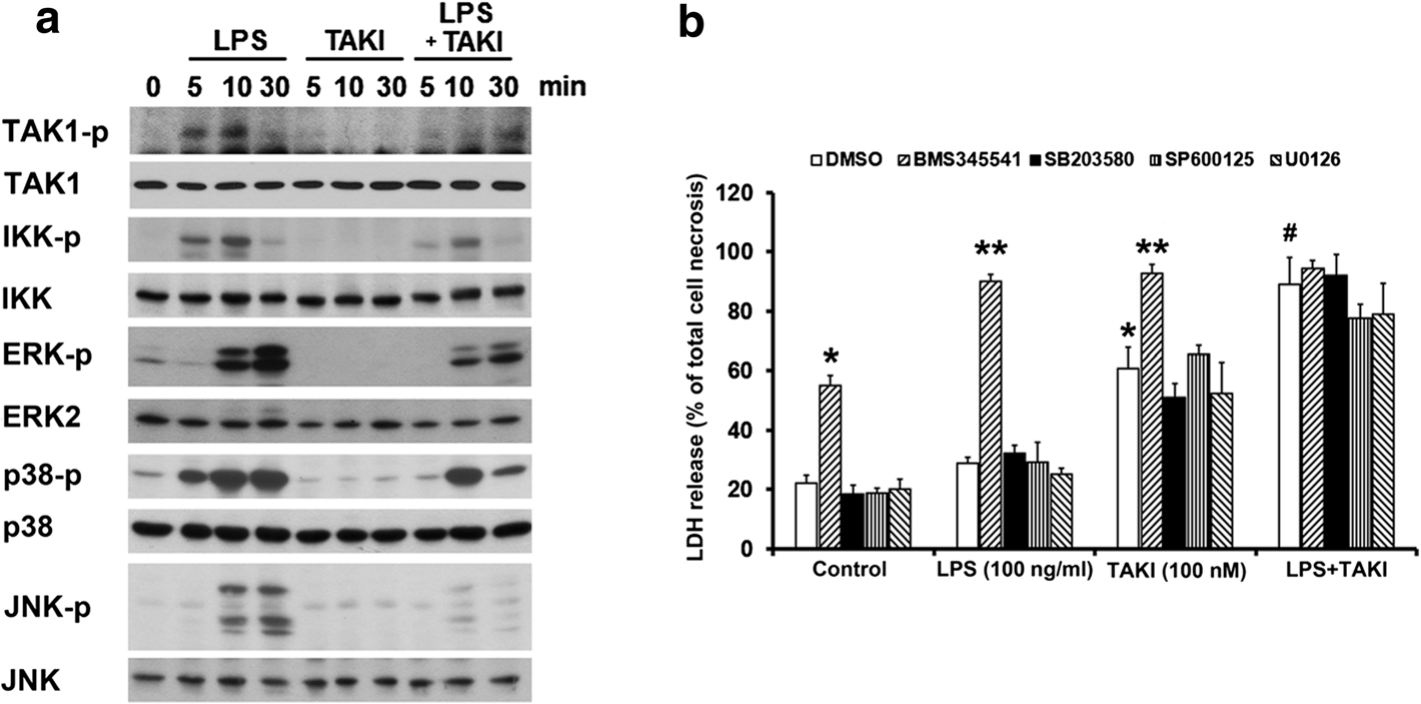
TAKI inhibits LPS-induced IKK, JNK, p38 and ERK activation. (**a**) BMDM were treated with LPS (100 ng/ml), TAKI (100 nM) or both for indicated time intervials, and then immunobloting against specific proteins was conducted. (**b**) BMDM were pretreated with BMS345541 (3 μM), SB230580 (10 μM), SP600125 (10 μM), or U0126 (10 μM) for 30 min, followed by LPS (100 ng/ml), TAKI (100 nM) or both for 6 h. The LDH activity released in the culture medium was then determined. *p<0.05, indicating significant effects of TAKI alone. #p<0.05, indicating the significant potentiation of TAKI-induced responses by LPS. **p<0.5, indicating significant potentiation of TAKI or LPS response by BMS345541

Apart from RIP1, we wonder the roles of MAPKs and IKK in TAKI-induced cell death. Immunobloting data revealed the abilities of TAKI to inhibit TAK1, IKK, ERK, p38 and JNK activation induced by LPS (Fig. 4a). Since TAKI can inhibit but not abolish IKK and MAPKs activation under LPS stimulation, we wondered if these kinases are involved to regulate cell death mechanisms under TAKI and/or LPS stimulation. Then we tested the effects of BMS345541, SB203580, SP600125, and U0126, inhibitors of IKK, p38, JNK and ERK, respectively, on TAKI-induced cell death. As shown in Fig. 4b, BMS345541 treatment alone can induce moderate cell death and this effect was further enhanced by LPS and TAKI. In contrast, MAPKs inhibitors failed to affect the death induced by TAKI, either in the presence or absence of LPS (Fig. 4b).

### TAKI-induced cell death involves mitochondria dysfunction

**Fig. 5:**
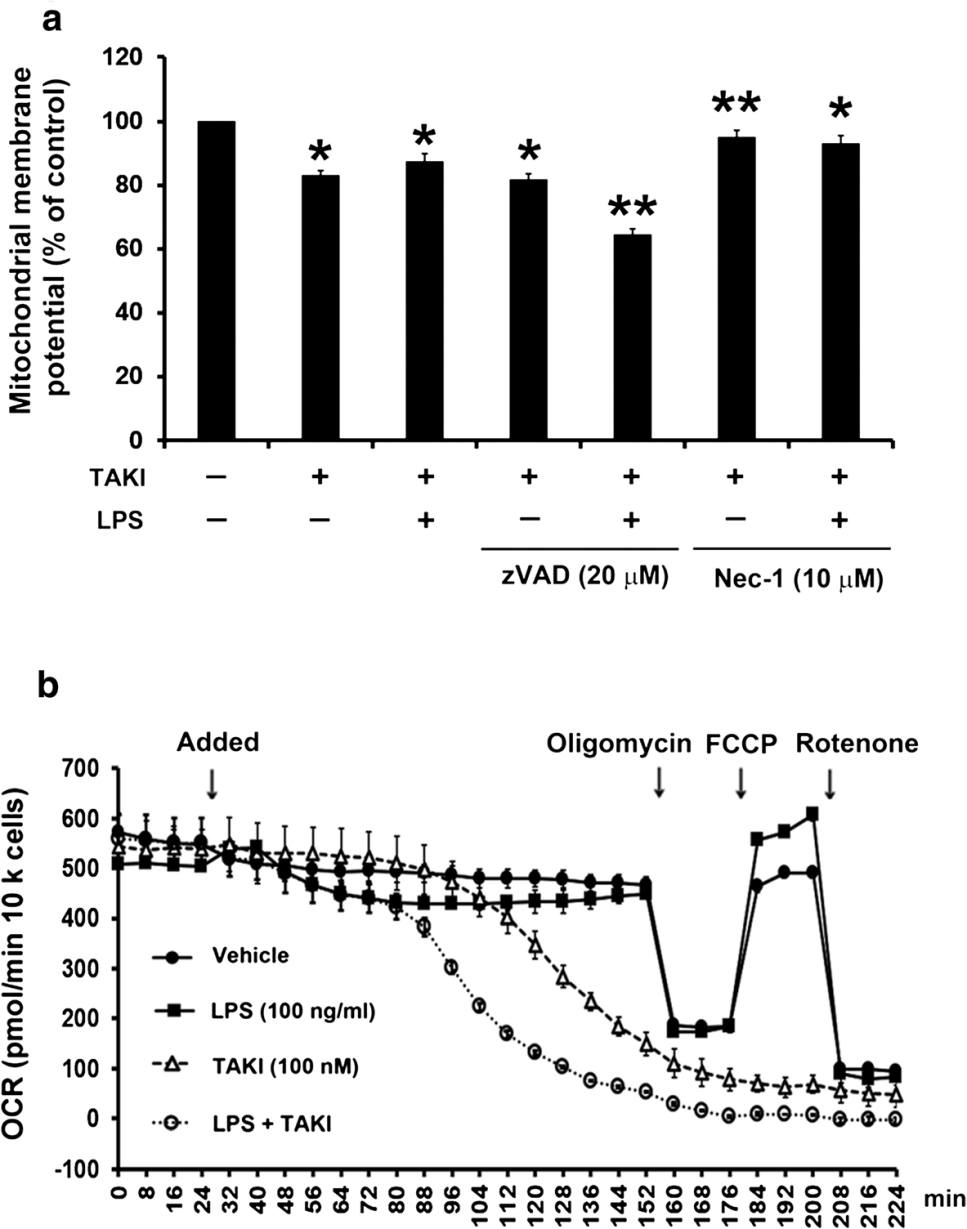
TAKI-induced cell death involves mitochondrial dysfunction. (**a**) BMDM were treated with 20 μM zVAD or 10 μM Nec-1 for 30 min, followed by TAKI (100 nM) and/or LPS (100 ng/ml) for 6 h. Then mitochondria membrane potential was detected. *p <0.05, indicating significant reduction of mitochondrial membrane potential caused by TAKI. **p <0.05, indicating significant potentiation or inhibiion of mitochondrial membrane potential loss by zVAD and Nec-1, respectively. (**b**) The mitochondrial metabolism was assessed by using the seahorse XF24 to measure the oxygen consumption rate (OCR). BMDM were treated with LPS (100 ng/ml), TAKI (100 nM), or LPS plus TAKI at 32 min as indicated with arrow. The inhibitor of ATP synthase, oligomycin (10 μg/ml) was added to discriminate ATP-linked respiration and proton leak. After adding an uncoupler of mitochondrial oxidative phosphorylation, FCCP (2.5 μM), OCR was raised to the maximal respiratory capacity. Finally rotenone (2.5 μM) was added to block the electron transport chain of oxidative phosphorylation

Since apoptosis is characterized as a consequence of mitochondrial dysfunction, we interested to understand if TAKI afffects the functions of mitochondria. First, we found that TAKI treatment caused the loss of mitochondrial membrane potential, which was unchanged by LPS, but was reversed by Nec-1 (Fig. 5a). Notably as the increased cell death response shown above, zVAD augmented mitochondrial membrane potential loss caused by TAKI/LPS (Fig. 5a). Next, we analyzed mitochondrial oxygen consumption rate (OCR) which reflects overall metabolic activity of mitochondria. As shown in Fig. 5b, TAKI treatment can gradually decrease OCR, while LPS itself did not induce significant OCR change. Nevertheless LPS co-treatment can accelerate the effect of TAKI. After drug treatment for 2 h, we added standard agents oligomycin, FCCP and rotenone to determine the mitochondrial respiration functions. We found oligomycin can rapidly decrease OCR levels in control and LPS groups, but not in TAKI and TAKI/LPS groups when their OCR level was markedly decreased. Meanwhile, unlike the general effects of FCCP and rotenone in control and LPS groups, both mitochondrial tested agents also cannot alter OCR in cells treated with TAKI, either with or without LPS (Fig. 5b).

### Intrinsic action of TNF-α contributes to TAKI-induced cell death

**Fig. 6:**
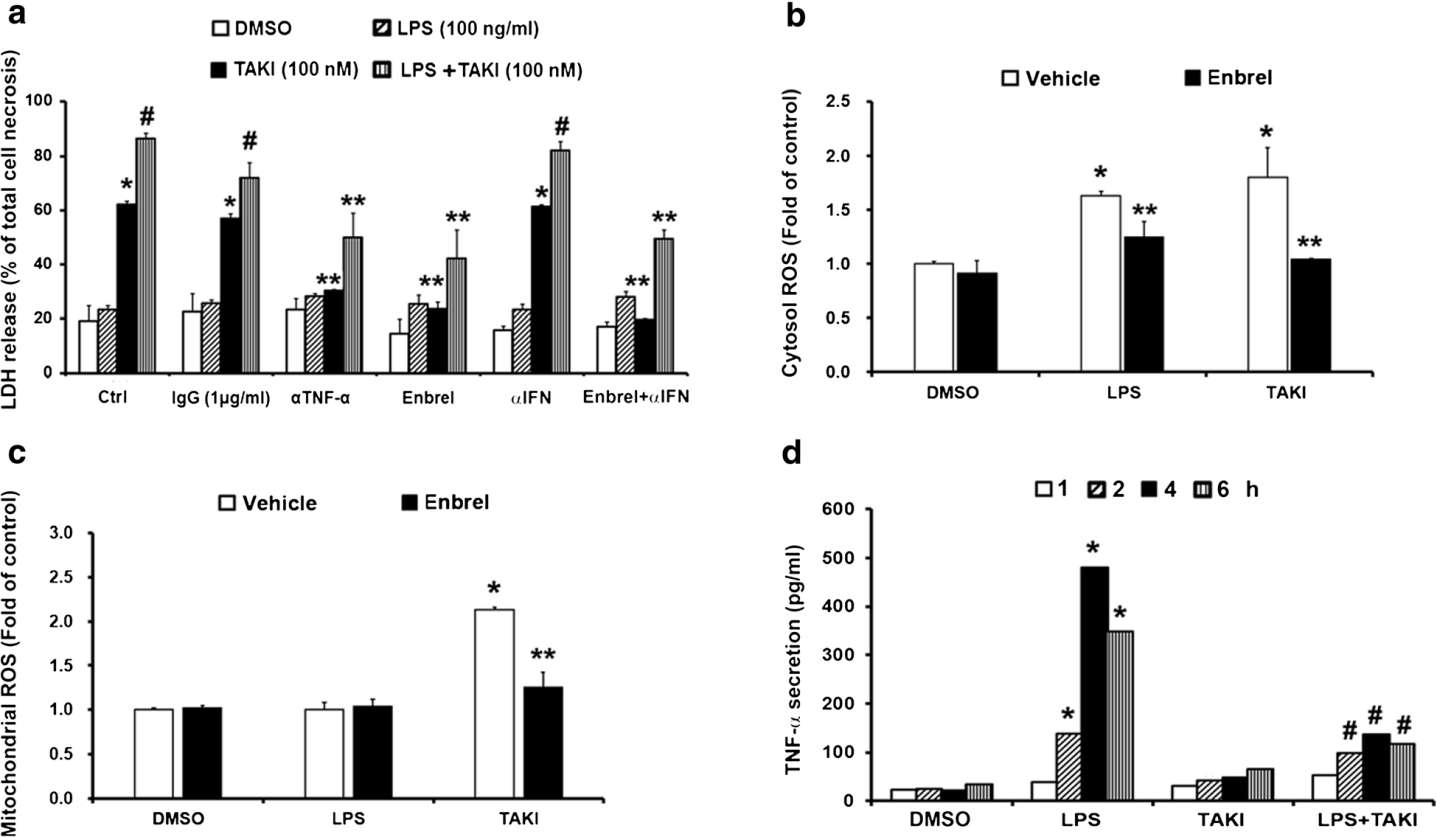
Intrinsic autocrine action of TNF-α contributes the TAKI-induced cell death. (**a**) BMDM were pre-treated with enbrel (10 μg/ml), control IgG (1 μg/ml), TNF-α Ab (1 μg/ml) or IFN-Ab (1 μg/ml) for 30 min, and then treated with TAKI (100 nM) and/or LPS (100 ng/ml) for 6 h. Cell culture medium was used to determine LDH release. (**b**, **c**) BMDM were pre-treated with enbrel (10 μg/ml) for 30 min followed by TAKI and LPS for indicated intervals, and then cytosolic ROS (**b**) and mitochondrial ROS (**c**) were determined. (**d**) BMDM were treated with LPS (100 ng/ml), TAKI (100 nM) or both for indicated time intervials, and then the medium was collected to determine TNF-α production. *p <0.05, indicating significant effects of TAKI and/or LPS. #*p* <0.05, indicating significant inhibition of LPS-induced TNF-α release by TAKI (**d**) or significant enhancement of TAKI-induced LDH release by LPS (**a**). **p <0.05, indicating significant inhibition of TAKI- and/or LPS-induced responses by enbrel or TNF-α Ab

Since TNF-α has been shown to induce cell death of various features depending on the cellular context, we wondered its involvement in TAKI-treated macrophages. Using neutralizing TNF-α antibody and enbrel (soluble TNF-α receptor), we found that the cell death induced by TAKI, regardless of the presence of LPS or not, was inhibited by both treatments (Fig. 6a). Accordingly enbrel can reduce cytosolic and mitochondrial ROS production caused by TAKI alone (Fig. 6b, c), and LPS-induced cytosolic ROS increase was also diminished by enbrel (Fig. 6b). These findings suggest that autocrine or paracrine action of TNF-α is involved in TAKI-induced ROS production and subsequent cytotoxicity. Next, the TNF-α production was determined by ELISA in order to correlate with cell death response. Surprisingly although TNF-α antibody and enbrel can abrogate TAKI-induced cell death and ROS production, no significant increase of TNF-α release under TAKI treatment was found. Stimulating macrophages with LPS as expected increased TNF-α production, and this effect was abrogated by the co-treatment with TAKI (Fig. 6d). Besides TNF-α, we also wondered the role of type I IFNs in cell death, as IFNs have been shown to regulate cell death in macrophages [36]. As shown in Fig. 6a, antagonistic IFN antibody had minimal effect on TAKI-induced cell death. These results all together suggest that the intrinsic and constitutive action of TNF-α is prerequisite for TAKI-induced ROS production and the subsequent cell death.

### TAKI-induced cell death exhibits the cell type specificity

**Fig. 7:**
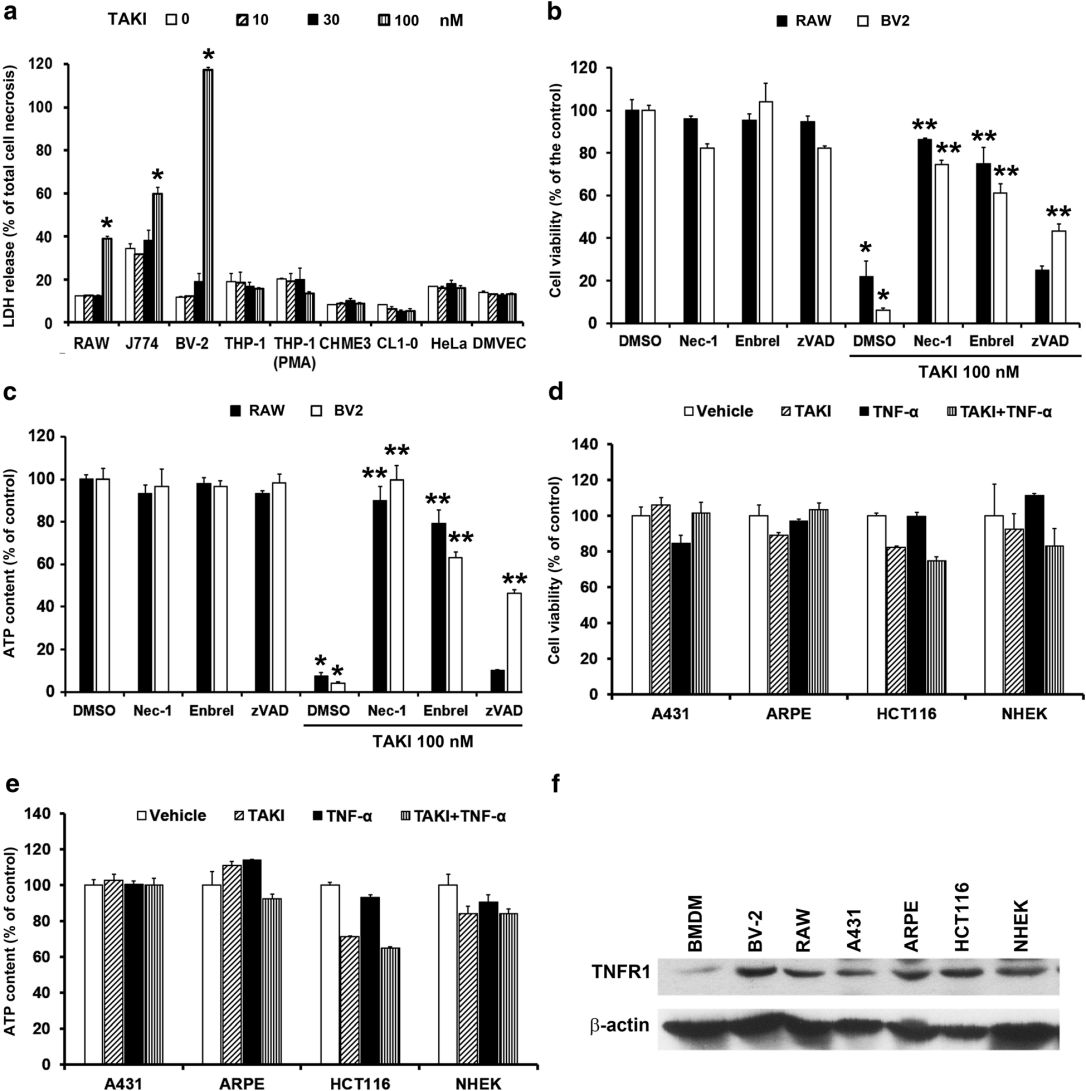
TAKI-induced cell death exhibits the cell type specificity, and less susceptibility is not due to deficiency of TNF-α action. (**a**) RAW264.7, J774, BV-2, THP-1 monocyte, THP-1 macrophage differentiated by PMA (10 nM, 24 h), CHME3, DMVEC, CL1.0 and HeLa were treated with the indicated concentrations of TAKI for 6 h. Then LDH release to culture medium was measured. (**b**, **c**) In RAW264.7 and BV-2 cells, necrostatin-1 (Nec-1, 10 μM), enbrel (10 μg/ml) or zVAD (20 μM) was treated for 30 min before the administration of TAKI (100 nM) for 6 h. (**d**, **e**) A431, ARPE, HCT116 and NHEK cells were treated with TAKI (100 nM) and TNF-α (10 ng/ml) for 6 h. MTT (**b**, **d**) and ATP assays (**c**, **e**) were conducted to measure cell viability. *p <0.05, indicating significant cell death induced by TAKI. **p <0.05, indicating significant inhibition of TAKI-induced cell death by Nec-1, enbrel or zVAD. (**f**) TNFR protein expression in various cell types was compared by immunobloting

Apart from BMDM, we also tested the effects of TAKI in other murine macrophages (RAW264.7 and J774) and microglial (BV-2) cell lines. Using necrotic marker LDH release as index, we found TAKI treatment also significantly induces cell death in these cell types with BV-2 displaying the prominent extent (Fig. 7a). Consistently Nec-1 and enbrel exerted the inhibitory effects on TAKI-induced cell death in terms of MTT (Fig. 7b) and ATP assays (Fig. 7c). Notably zVAD treatment can reverse the cytotoxicity of TAKI in BV-2 cells, but not in RAW264.7 cells. In order to understand the cytotoxicity effect of TAKI is general or not, we examined several cell types. We found that TAKI at concentrations up to 100 nM failed to alter the cell viability in human THP-1 monocytes, THP-1-derived macrophages, and CHME, 3 microglia. Neither normal human epidermal keratinocyte (NHEK), retinal pigment epithelial cells (ARPE), dermal microvascular endothelial cells (DMVEC) or cancer cells like A431 squamous cell carcinoma, CL1.0 lung cancer, HeLa cervical cancer, and HCT116 colon cancer was affected by TAKI (Fig. 7a, d, e). To clear if less intrinsic TNF-α action made such susceptibility difference, we co-treated TNF-α (10 ng/ml) together with TAKI in these non-responsive cell types. As a result, TAKI still cannot induce cytotoxicity upon TNF-α co-treatment by either MTT (Fig. 7d) or ATP assays (Fig. 7e). After observing this, we wondered if distinct expression level of endogenous TNFR determines the susceptibility in response to TAKI + TNF-α. We thus determined the basal level of TNFR in these cell types. Immunobloting data revealed that BV-2, HCT116 and NHEK expressed higher and comparatively equivalent level of TNFR1, RAW264.7, A431 and ARPE expressed moderate amount, and BMDM expressed the lowest amount (Fig. 7f). Since TNFR1 expression level does not correlate to the susceptibility of TNF-α-dependent cytotoxicity, we suggest it is the cellular context rather than TNF-α signaling determines the cell viability in response to TAKI.

## Discussion

Given that TAK1 exerts multifaceted functions in controlling cell fate and inflammatory responses, targeting TAK1 for inflammatory disorders and cancer has raised great attention. The therapeutic potential of TAKI in enhanced chemotherapeutic efficacy might result from its potent inhibition of the IKK-NF-κB pathway, potentiation of ROS and caspase activity [37–39]. In this aspect, previous studies demonstrated the effectiveness of TAK1 inhibition or deficiency in skin cancer [40], multiple lymphoma [41], KRAS-dependent colon cancers [42], breast cancer [37] and cervical cancer [38]. Moreover, a TAKI closely analog, E6201 is currently in phase II clinical trials for the treatment of melanoma [43], and may be useful for psoriasis [44] and arthritis [45]. On the other hand, the neuroprotective effects of TAK inhibition for stroke, traumatic brain injury and neurodegenerative diseases are associated with a reduced activation of JNK that leads to inflammation and apoptosis [46–48].

According to its multifaceted signaling functions as mentioned above, TAK regulation of cell viability is believed to be cell type and cell context dependent. Since macrophages play a major role in acute inflammatory responses in various tissues and diseases [49], and TAK1 knockout leads to the automatic macrophage death [2, 50, 51], in this study we used macrophages as the cell model to deep in the death regulating mechanisms and signaling pathways controlled by TAK1. Our present data showed that TAKI can induce a dramatic cell death in primary BMDM. Different from previous finding showing the delayed onset of spontaneous cell death in TAK1^−/−^ BMDM (around 45 % cell death at 4 day) [50]. TAKI-induced cell death in BMDM is of rapid onset (around 60 % cell death at 6 h treatment). Currently we do not have clear explanation for this difference, but effects of TAK1 gene deletion on *in vitro* macrophage differentiation and/or some cellular adaptive regulation cannot be excluded. In our study, we found the death caused by TAKI in BMDM is of caspase-dependent apoptosis mode. The supporting evidence of this notion includes the appearance of apoptotic landmark Annexin V (Fig. 1c), and activation of caspases 8 and 3 (Fig. 2a). Here we rule out the involvement of LC3 dependent autophagy or PARP1 dependent cell death mode (i.e. parthanatos) in this action, because TAKI cannot trigger LC3II increase (Fig. 2a) and its induced cell death is not affected by PARP1 inhibitor (data not shown). So our result is in agreement with previous report showing apoptosis occurring in TAK1^−/−^ BMDM [50]. A study reported by Morioka et al. also showed that ablation of TAK1 in MEF led to caspase-dependent apoptosis [24].

This study for the first time shows the ability of TAK inhibitor alone to induce RIP1-dependent apoptosis, unlike the case requiring exogenous TNF-α to trigger RIP1-dependent apoptosis in MEF with TAK1-deficient or inhibition condition [52]. Another interesting finding of our study is that constitutive TNFR signal through autocrine loop plays an important role to control the death action of TAKI in BMDM, RAW264.7 macrophages and BV-2 microglia. Although TAKI alone is not able to change basal TNF-α production (Fig. 6d), TAKI-induced ROS production (Fig. 6b, c) and cell death (Fig. 6a) were abrogated by enbrel and neutralizing antibody of TNF-α. These findings indicate the crucial determinant of autocrine factor TNF-α to timely control the cell fate in macrophages and microglia. Even lower amount of TNF-α secreted at resting state is sufficient to render such death regulation action. Indeed as previously reported, depending on the statuses of caspase, RIP1 and TAK1 activity, exogenous TNF-α can mediate cell survival or various modes of cell death [2]. Thus our current findings support the complexity of TNFR signaling pathways in coordination with TAK1 activity to timely control the cell fate. Moreover, we for the first time to strengthen the key role of constitutive and autocrine action of TNF-α in controlling macrophages and microglia survival. Such intrinsic and quiescent function of TNF-α can be unmasked and become sensitized to impact cellular response when TAK1-dependent protection signals and functions are severely impaired.

In this study using TAK inhibitor we demonstrated the cell type specificity for TAKI-induced cytotoxicity. We found TAKI itself can induce cell death in murine macrophages (BMDM, RAW264.7, J774) and microglia (BV-2), but not in cancer cells (CL1.0, HeLa, HCT116, A431), human THP-1 monocytes, human CHME3 microglia, and primary normal cells (NHEK, ARPE and DMVEC) (Fig. 7). These data strongly suggest that TAK-dependent cell signaling and molecular events in regulating cell viability are cell type specific. In addition, although TNFR signaling as discussed above is important to mediate TAKI-induced cell death in BMDM, RAW264.7 and BV-2, it is not the major factor to explain why there is no death response to TAKI in other cell types. This is because constitutive TNFR1 level in various cell types does not correlate to the cell death susceptibility in response to TAKI plus TNF-α (Fig. 7a, e, f). Although TNFR1 rather than TNFR2 is known as the major receptor for TNF-α-induced signaling and cellular functions, our current study still cannot exclude the potential role of TNFR2 in controlling the cell viability of macrophages and microglia in response to TAKI. Therefore, it is the distinct cellular context rather than TNF-α signals via TNFR1 alone to determine the cell susceptibility to TAKI.

Previous studies conducting in TAK1 deletion cell models have demonstrated the role of ROS accumulation for cell death [53, 54]. In our study using TAKI we similarly observed the increased cytosolic and mitochondrial ROS production from TNFR signals in BMDM (Fig. 6b, c). Moreover, RIP1 inhibition by necrostatin-1 only reduces mitochondrial but not cytosolic ROS elevation (Fig. 3c, d). TNFR1 activation has been shown to activate NADPH oxidase for cytosolic ROS production, which causes cIAP protein depletion and activation of RIP1-dependent caspase-8 [2]. Besides activating caspase-8, RIP1 activation also leads to ROS production in mitochondria [52]. Based on these findings we suggest that TAK inhibition can unmask intrinsic TNFR signal for the rapid initiation of cytosolic ROS production, which in turn triggers RIP1 activation and late mitochondrial ROS production. Accordingly we suggest the enhanced cell death upon LPS co-treatment is through increased RIP1-dependent mitochondrial ROS production (Fig. 3b). Previously TLR4 activation by LPS was shown to activate RIP1 through TRIF signaling pathway [55]. Similar to TNF-α, NO has been shown to induce programmed cell death in endothelial cells, and both RIP1 and ROS are involved [56]. Herein, we explored the possible role of NO in TAKI-elicited cell death in BMDM. We found that treatment with iNOS inhibitor L-NAME did not affect cell death induced by TAKI, either in the presence or absence of LPS (data not show). In addition, our data suggest the death mechanism caused by TAKI is independent of IKK, because we observed the additive cytotoxicity between TAKI and IKK inhibitor (Fig. 4b).

Depending on the cell types, caspase inhibition has been shown to switch cell death from apoptosis to either autophagic cell death or necroptosis [14, 32–34, 57]. Notably the induction of necroptosis by caspase inhibitor in macrophages and MEF is also RIP1- and TNFR-dependent [14, 32–34]. In our study, zVAD treatment cannot block TAKI-induced cell apoptosis in BMDM and RAW264.7 macrophages (Fig. 7b, c), but an even more increased cell death was observed in BMDM (Fig. 2c). Such cell death under zVAD treatment in BMDM still depends on RIP1 (Fig. 2b, c). Therefore we suggest zVAD co-treatment can drive cells proceeding a RIP1-dependent but caspase-independent apoptosis. Supporting this suggestion, under caspase inhibition mitochondrial cell death resulting from loss of respiration, but not from cytotoxic protein release has been reported [58]. Therefore it is still necessary to explore the death mechanism in details under TAKI + zVAD treatment. It is also needed to elucidate the mechanisms underlying the partial protective effect of zVAD in TAKI-treated BV-2 cells, which is unlike the effects seen in BMDM and RAW264.7 cells. Moreover whether autophagy is involved in TAKI + zVAD-induced cell death in BMDM needs further investigation.

Taken together, we for the first time demonstrate TAKI-induced cytotoxicity is cell context specific and depends on the endogenously constitutive TNFR signaling. An IKK-independent cell survival pathway downstream of TAK1 is essential for macrophages to prevent apoptosis signal induced by autocrine TNF-α through RIP1-ROS-caspase pathway. All these results highlight the complexity of cell death regulation in macrophages in viewing the functional balance of molecules among TNFR, TAKI, RIP1 and caspases. Notably, since TAK inhibition is under drug development for sensitizing chemotherapy in cancer patients and for several inflammation-associated diseases, our current findings are warrant in considering the pros and cons for targeting TAK in disease treatment.

## Conclusion

TAK1 is involved to regulate cell survival. Inhibition of TAK1 leading to cell death is cell context specific. In murine macrophages, TAKI-induced apoptosis is dependent on the constitutive autocrine action of TNF-α, RIP1activation and ROS production.

## Abbreviations

AP-1: Activator protein-1
ARPE: Retinal pigment epithelial cells
BHA: Butylated hydroxyanisol
BMDM: Bone marrow-derived macrophages
DCFH-DA: 2′,7′-Dichlorodihydrofluorescein diacetate
DMEM: Dulbecco’s modified Eagle’s medium
DMVEC: Dermal microvascular endothelial cells
FCCP: Carbonyl cyanide p-(trifluoromethoxy) phenylhydrazon
FMK: Fluoro-methylketone
IL: Interleukin
LDH: Lactic dehydrogenase
LPS: Lipopolysaccharide
MEF: Mouse embryonic fibroblasts
MTT: 3-(4,5-Dimethylthiazol-2-yl)-2,5-diphenyltetrazolium bromide
NAC: N-Acetyl-L-cysteine
Nec-1: Necrostatin-1
NHEK: Normal human epidermal keratinocytes
OCR: Oxygen consumption rate
PI: Propidium iodide
RIP: Receptor-interacting protein
ROS: Reactive oxygen species
TAK1: Transforming growth factor-β (TGF-β)-activated kinase 1
TAKI: 5Z-7-Oxozeaenol
TLR: Toll-like receptor
TNF: Tumor necrosis factor
TNFR1: TNF receptor 1
zVAD: Benzyloxycarbonyl-Val-Ala-Asp
